# The Aspergillus fumigatus Phosphoproteome Reveals Roles of High-Osmolarity Glycerol Mitogen-Activated Protein Kinases in Promoting Cell Wall Damage and Caspofungin Tolerance

**DOI:** 10.1128/mBio.02962-19

**Published:** 2020-02-04

**Authors:** Eliciane Cevolani Mattos, Lilian Pereira Silva, Clara Valero, Patrícia Alves de Castro, Thaila Fernanda dos Reis, Liliane F. C. Ribeiro, Mark R. Marten, Rafael Silva-Rocha, Cauã Westmann, Carlos Henrique Tomich de Paula da Silva, Carlton Anthony Taft, Narjes Al-Furaiji, Michael Bromley, Uffe H. Mortensen, J. Philipp Benz, Neil Andrew Brown, Gustavo H. Goldman

**Affiliations:** a Faculdade de Ciências Farmacêuticas de Ribeirão Preto, Universidade de São Paulo, Ribeirão Preto, Brazil; b University of Maryland Baltimore County (UMBC), Department of Chemical, Biochemical and Environmental Engineering, Baltimore, Maryland, USA; c Faculdade de Medicina de Ribeirão Preto, Universidade de São Paulo, Ribeirão Preto, Brazil; d Centro Brasileiro de Pesquisas Físicas (CBPF), Rio de Janeiro, Brazil; e Division of Infection, Immunity and Respiratory Medicine, School of Biological Sciences, Faculty of Biology, Medicine and Health, University of Manchester, Manchester, United Kingdom; f Eukaryotic Molecular Cell Biology, Section for Synthetic Biology, Department of Biotechnology and Biomedicine, Technical University of Denmark, Kongens Lyngby, Denmark; g Holzforschung München, TUM School of Life Sciences Weihenstephan, Technical University of Munich, Freising, Germany; h Institute for Advanced Study, Technical University of Munich, Garching, Germany; i Department of Biology and Biochemistry, University of Bath, Bath, United Kingdom; j Departamento de Química, Faculdade de Filosofia, Ciências e Letras de Ribeirão Preto (FFCLRP), Universidade de São Paulo, Ribeirão Preto, Brazil; Duke University Medical Center

**Keywords:** *Aspergillus fumigatus*, cell wall integrity pathway, MpkC, SakA, caspofungin, osmotic stress

## Abstract

The filamentous fungus Aspergillus fumigatus can cause a distinct set of clinical disorders in humans. Invasive aspergillosis (IA) is the most common life-threatening fungal disease of immunocompromised humans. The mitogen-activated protein kinase (MAPK) signaling pathways are essential to the adaptation to the human host. Fungal cell survival is highly dependent on the organization, composition, and function of the cell wall. Here, an evaluation of the global A. fumigatus phosphoproteome under cell wall stress caused by the cell wall-damaging agent Congo red (CR) revealed 485 proteins potentially involved in the cell wall damage response. Comparative phosphoproteome analyses with the Δ*sakA*, Δ*mpkC*, and Δ*sakA* Δ*mpkC* mutant strains from the osmotic stress MAPK cascades identify their additional roles during the cell wall stress response. Our phosphoproteomics allowed the identification of novel kinases and transcription factors (TFs) involved in osmotic stress and in the cell wall integrity (CWI) pathway. Our global phosphoproteome network analysis showed an enrichment for protein kinases, RNA recognition motif domains, and the MAPK signaling pathway. In contrast to the wild-type strain, there is an overall decrease of differentially phosphorylated kinases and phosphatases in Δ*sakA*, Δ*mpkC*, and Δ*sakA* Δ*mpkC* mutants. We constructed phosphomutants for the phosphorylation sites of several proteins differentially phosphorylated in the wild-type and mutant strains. For all the phosphomutants, there is an increase in the sensitivity to cell wall-damaging agents and a reduction in the MpkA phosphorylation upon CR stress, suggesting these phosphosites could be important for the MpkA modulation and CWI pathway regulation.

## Abstract (precis)

Aspergillus fumigatus is an opportunistic human pathogen causing allergic reactions or systemic infections, such as invasive pulmonary aspergillosis in immunocompromised patients. The mitogen-activated protein kinase (MAPK) signaling pathways are essential for fungal adaptation to the human host. Fungal cell survival, fungicide tolerance, and virulence are highly dependent on the organization, composition, and function of the cell wall. Upon cell wall stress, MAPKs phosphorylate multiple target proteins involved in the remodeling of the cell wall. Here, we investigate the global phosphoproteome of the Δ*sakA* and Δ*mpkC* A. fumigatus and high-osmolarity glycerol (HOG) pathway MAPK mutants upon cell wall damage. This showed the involvement of the HOG pathway and identified novel protein kinases and transcription factors, which were confirmed by fungal genetics to be involved in promoting tolerance of cell wall damage. Our results provide understanding of how fungal signal transduction networks modulate the cell wall. This may also lead to the discovery of new fungicide drug targets to impact fungal cell wall function, fungicide tolerance, and virulence.

## INTRODUCTION

Aspergillus fumigatus is a filamentous fungus that can cause disease in humans (1). Depending on a patient’s immunological status, A. fumigatus can cause a distinct set of clinical disorders that extend from severe allergies to lethal disseminated infections (1). Invasive aspergillosis (IA) is the most common life-threatening fungal disease in immunocompromised humans, and mortality rates can reach 90% (2–6). Disseminated fungal infections are treated with antifungal drugs, including polyenes, azoles, and echinocandins (7). However, infections by antifungal drug-resistant pathogens are on the rise, presenting severe treatment constraints (8). Several factors are important for A. fumigatus infection and survival in the human host, including hypoxia resistance, iron assimilation, gliotoxin production (depending on the immune status of the host), presence of dihydroxynaphthalene (DHN) melanin, and thermophily (9–19). How these genetic traits are integrated in response to environmental cues is poorly understood; thus, a better understanding of stress response signaling networks involved in virulence is essential for the development of improved IA treatments.

The highly conserved mitogen-activated protein kinase (MAPK) signaling pathways are essential for adaptation to environmental changes (20, 21). The MAPK cascades are important for relaying, integrating, and amplifying intracellular signals and are crucial signaling components involved in many cellular processes (20, 21). In filamentous fungi, the conserved cell wall integrity (CWI), pheromone response/filamentous growth, high-osmolarity glycerol (HOG), and MAPK pathways have been shown to influence numerous virulence traits, including invasive growth, biofilm formation, mycotoxin production, and antifungal tolerance (22–24).

A. fumigatus has four MAPKs: (i) MpkA is the central regulator of CWI pathway and also plays a role in oxidative stress tolerance (25, 26), and several other cellular processes (27); (ii) MpkB regulates the pheromone response/filamentous growth pathway, which is important for conidiation and dihydroxynaphthalene (DHN) melanin production (28); and (iii) SakA and MpkC are paralogues that constitute the main regulators of the HOG pathway (29). The MpkC and SakA protein sequences are very similar, and they play a role in caspofungin tolerance and carbon source utilization, respectively (25, 30, 31). In Aspergillus nidulans and A. fumigatus, MpkC and SakA physically interact, but also show distinct and shared functions during stress responses and development (32–34). However, little is known about the mechanisms by which MpkC and SakA execute their signaling functions.

Fungal cell survival is highly dependent on the organization, composition, and function of the cell wall. The A. fumigatus cell wall is essential for cell shape, virulence, and prevention of cell lysis due to strong osmotic pressures. In addition, it plays a critical role in adhesion, host recognition, and evasion of the mammalian immune system (35–37). Although the cell wall is rigid, it is also a dynamic structure and is constantly being remodeled during fungal growth and development (38–40). Echinocandins, which inhibit the biosynthesis of the cell wall component β-1,3-glucan, are among the most recent antifungal drugs to be developed (3) and highlight that cellular integrity is a good target for further development of antifungals. Cell wall biosynthesis and the CWI pathway therefore provide excellent targets for further antifungal drug development.

The A. fumigatus CWI pathway has already been characterized, and it is mainly composed of the PkcA-MpkA-Rlm1 module (36, 41–45). Recently, we demonstrated that the HOG MAPs SakA and MpkC are independently required to mount an adequate response to cell wall damage (29). The Δ*sakA* and the double Δ*sakA* Δ*mpkC* mutants were more sensitive to osmotic and oxidative stresses and to cell wall-damaging agents. Both MpkC:GFP (green fluorescent protein) and SakA:GFP translocated to the nucleus upon osmotic and cell wall stress, with SakA:GFP showing a quicker response. The phosphorylation of MpkA from the CWI pathway in response to osmotic and cell wall stress was dependent on MpkC and SakA, suggesting that both the CWI and HOG pathways collaborate upon exposure to several types of stresses and during cell wall biosynthesis (29, 34).

“Omics” high-throughput techniques such as genomics, transcriptomics, metabolomics, and proteomics have revolutionized biological research. Phosphoproteomics is a branch of proteomics important for the characterization of proteins that are modified posttranslationally by the addition of a phosphate group. Phosphorylation is a reversible protein modification that can regulate cell signaling networks through the regulation of protein function, subcellular localization, complex formation, and protein degradation (46). It is estimated that 30% to 65% of all proteins may be phosphorylated (46, 47). Phosphoproteomics, in particular, has been utilized to elucidate novel signaling mechanisms (48). Phosphoproteomics provides information about which protein or pathway might be activated, since generally there is a correlation between a change in protein phosphorylation and protein activity. Phosphoproteomics has been used for understanding mitotic exit and cell wall integrity pathways in Saccharomyces cerevisiae (49, 50); hyphal morphogenesis in Candida albicans (51); asexual development and multiple stress responses in Beauveria bassiana (52); cellular processes involved in morphology, secretion, protein kinase A regulation, and catabolite repression in A. nidulans (53, 54); and the aflatoxin production in Aspergillus flavus (55). Here, we evaluate the global A. fumigatus phosphoproteome exposed to cell wall-damaging agents, identifying 485 proteins putatively involved in the cell wall stress response. Comparative phosphoproteome analyses of the wild-type (WT) and Δ*sakA*, Δ*mpkC*, and Δ*sakA* Δ*mpkC* mutant strains showed the involvement of the HOG pathway and identified novel protein kinases and transcription factors, which were confirmed by fungal genetics to be involved in promoting CWI signaling and tolerance of cell wall damage.

## RESULTS

### A. fumigatus phosphoproteome upon cell wall damage.

**FIG 1:**
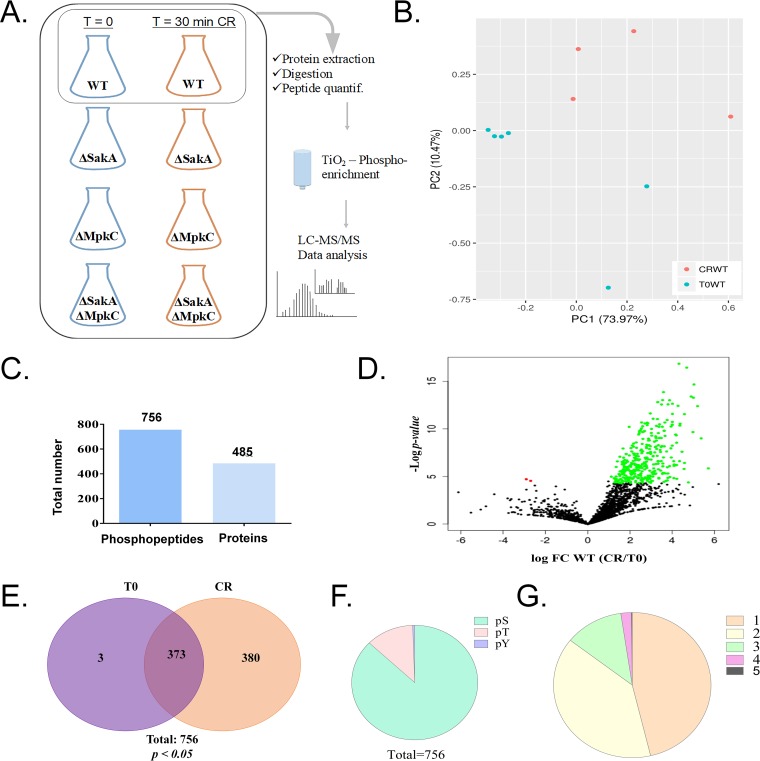
Phosphorylation profile of proteins from A. fumigatus wild type upon exposure to Congo red (CR 10 min). (A) Workflow of experimental design for phosphoproteome results. (B) Principal-component analysis plot for the A. fumigatus wild type without CR treatment (T = 0, T0WT; six replicates) and with 10-min CR treatment (CRWT; four replicates). (C) Number of phosphopeptides and unique proteins modified by phosphorylation during CR treatment. (D) Volcano plot of total phosphopeptides identified, showing a major increase of phosphorylation after CR treatment. FC, fold change. (E) Venn diagram of total 756 phosphopeptides indicating the profile of total phosphorylation (380) or total dephosphorylation (3) in wild-type strains. (F) Number of phosphosites in serine (S), threonine (T), or tyrosine (Y) for total phosphopeptides. (G) Number of phosphorylation sites for each peptide.

A phosphoproteomics study was used as a first step to understand how protein phosphorylation is involved in the response to the cell wall damage. To achieve this, mycelia were collected from liquid minimal medium before (T0) and after adding 200 mM Congo red (CR) for 30 min (CR30). From the wild-type, Δ*sakA*, Δ*mpkC*, and Δ*sakA* Δ*mpkC* strains, at T0 and CR30, total protein was extracted, trypsin digested, subjected to TiO_2_ phosphopeptide enrichment, and analyzed by liquid chromatography-tandem mass spectrometry (LC-MS/MS), as illustrated in Fig. 1A.

Principal-component analyses of the wild-type phosphoproteome showed the cluster of untreated samples separated from those exposed to CR (Fig. 1B). A total of 4,499 phosphopeptides were identified with “localization probabilities” higher than 0.75 (75% [see Table S1A in the supplemental material]). A total of 756 phosphopeptides were found to be significantly different between the untreated T0 and the CR-treated CR30 sample (Student *t* test, *P < *0.05), corresponding to 485 proteins (Fig. 1C). Among these phosphopeptides, three were identified only in T0 samples and 380 were exclusive to CR-treated samples while 373 were observed in both treatments (Fig. 1E and Table S1B). The distribution of phosphosites according to amino acid residues followed a pattern typical of eukaryotic organisms, with 99% occurring on serine and threonine and <1% on tyrosine residues (Fig. 1F and Table S1B). Approximately half of the phosphoproteins contained a single phosphorylation site, and phosphoproteins containing one to three phosphosites accounted for the vast majority of phosphoproteins detected (Fig. 1G and Table S1B).

10.1128/mBio.02962-19.3 TABLE S1 (A) Complete list of phosphosites identified in the wild-type strain in the presence of Congo red. (B) List of phosphosites identified in the wild-type strain in the presence of Congo red with *P* < 0.05. (C) List of protein kinases observed as phosphorylated in the presence of Congo red in the wild-type strain. (D) List of transcription factors observed as phosphorylated in the presence of Congo red in the wild-type strain. (E) Enrichment for functional protein association networks. Download Table S1, XLSX file, 2.5 MB. Copyright © 2020 Mattos et al. 2020 Mattos et al. This content is distributed under the terms of the Creative Commons Attribution 4.0 International license.

**FIG 2:**
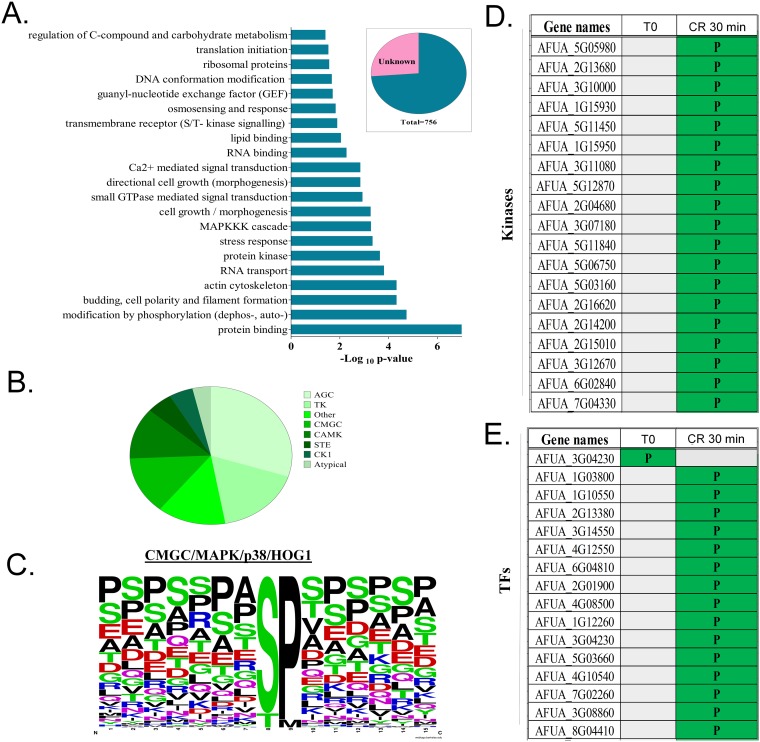
Functional categorization and identification of kinase substrates. (A) Phosphopeptide functional categorization of approximately 75% of proteins with known function. (B) Profile of distribution of kinase families that are able to phosphorylate the 756 phosphopeptides identified by LC-MS/MS as predicted by Group-based Prediction System 3.0 (GPS; with medium-stringency cutoff). (C) Consensus sequence of the site of phosphorylation of p38/Hog1 peptide substrates predicted by Group-based Prediction System 3.0 (GPS; with medium-stringency cutoff). (D and E) Phosphorylation distribution of all kinases (D) and transcription factors (TFs) (E) modulated by phosphorylation during Congo red response. Gray blocks indicate lack of the phosphopeptide, while green (P) indicates the presence of a phosphopeptide.

FunCat (https://sbi.hki-jena.de/fungifun/fungifun.php) analyses for the 485 proteins showed an enrichment for proteins involved in the regulation of C-compound, translation initiation, osmosensing and response, Ca^2+^-mediated signal transduction, MAPK kinase kinase (MAPKKK) cascades, stress responses, encoded protein kinases, actin cytoskeleton, and budding cell polarity and filament formation (Fig. 2A). This implies that the phosphorylation response to cell wall damage involves stress responses, translation, protein kinases, and different MAPK and calcium-mediated signaling mechanisms.

Possible kinase groups targeting each of the 756 phosphorylation sites were predicted by Group-based Prediction System 3.0 (GPS; with medium-stringency cutoff) (Fig. 2B). Based on kinase-substrate relationships, seven serine/threonine kinase families accounted for most of the identified phosphorylation sites (Fig. 2B). The most frequent serine/threonine kinase families (more than 50%) were AGC, TK, and atypical, followed by the CMGC family (15%), which includes MAPKs, such as the HOG (p38) MAPK (Fig. 2B). For example, a frequently observed phosphoserine/threonine kinase was p38 (CMGC/MAPK/p38/Hog) (Fig. 2C). In fact, the analysis of the substrate sequences phosphorylated by p38 MAPKs indicates a high conservation of a serine (S) and a proline (P) in positions +8 and +9 (>90%) among the putative targets of p38 MAPK (Fig. 2C).

### Protein kinases and transcription factors involved in the cell wall damage.

**FIG 3:**
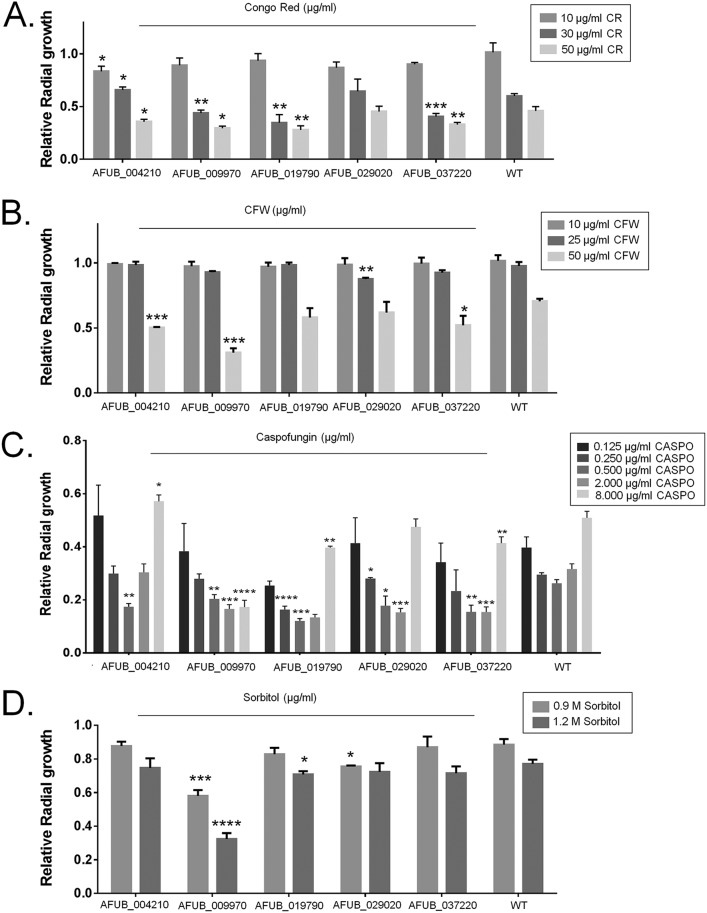
Phosphoproteomics allows the identification of novel transcription factors involved in the CWI pathway. A. fumigatus conidia (1 × 10^5^) were inoculated on solid minimal medium (MM) with different concentrations of Congo red (CR), calcofluor white (CFW), caspofungin (CASPO), and sorbitol and grown for 5 days at 37°C. All plates were grown in triplicate, and the average ± SD was plotted. A Student *t* test using Prism GraphPad (version 6) was applied to confirm the statistical significance difference between treatment and control (*P* value < 0.05, ***; < 0.01, **; < 0.001, *).

We have visually inspected and identified the phosphopeptides that corresponded to the 32 protein kinases and 27 transcription factors (TFs) identified with increased phosphorylation upon CR exposure (Fig. 2D and E; Table S1C and D). Eleven candidate protein kinase and 5 TF-encoding genes were randomly selected (Table 1), and it was verified whether their corresponding single-gene deletion mutants were more sensitive to cell wall-damaging agents CR, calcofluor white (CFW), and caspofungin and to high-sorbitol-induced osmotic stress. No significant growth differences were observed among these protein kinase null mutants compared to the wild-type strain (Fig. S1A to D). However, the TF null mutants lacking the gene AFUB_004210, AFUB_009970, AFUB_019790, or AFUB_037220 were more sensitive to CR (Fig. 3A), while mutants lacking AFUB_004210, AFUB_009970, and AFUB_037220 were more sensitive to calcofluor white (CFW), than the wild-type strain (Fig. 3B). All the TF null mutants showed some extent of increased sensitivity to caspofungin (Fig. 3C). The TF null mutants for AFUB_009970 and AFUB_019790 were also more sensitive to osmotic stress (Fig. 3D).

**TABLE 1:** Genes encoding protein kinases and transcription factors that showed phosphorylation when the wild-type strain is exposed to Congo red for 30 min

Protein	Putative function
Protein kinases	
HriA (AFUB_032300)	Eukaryotic translation initiation factor 2 alpha kinase activity and role in G_1_ cell cycle arrest in response to nitrogen starvation, negative regulation of cytoplasmic translational initiation in response to stress
SrrB (AFUB_030660)	Response regulator of a two-component phosphorelay system
Kin1 (AFUB_010510)	Serine/threonine protein kinase involved in regulation of exocytosis
SnfA (AFUB_018770)	Ortholog(s) has AMP-activated protein kinase activity, ADP ribosylation factor guanyl-nucleotide exchange factor activity
CmkA (AFUB_029320)	Calcium/calmodulin-dependent protein kinase
Prr1 (AFUB_029820)	Serine/threonine protein kinase; inhibits pheromone-induced signaling downstream of MAPK, possibly at the level of the Ste12p transcription factor
PkhA (AFUB_036500)	Phosphoinoside-dependent protein kinase
CmkC (AFUB_053520)	Calmodulin-dependent protein kinase activity
KfsA (AFUB_014350)	Checkpoint kinase; required for normal septation in conidiophores, not hyphae; present in hyphae and conidiophores at septa and cortex, not at spindle pole bodies
Oca2 (AFUB_059390)	Role in negative regulation of transcription by RNA polymerase II, regulation of nitrogen utilization, response to salt stress
FpkA (AFUB_059090)	Ribosomal S6 kinase (RSK)
Transcription factors	
AFUB_004210	Unknown function
AFUB_009270	Unknown function
AsgA (AFUB_019790)	Zinc cluster protein proposed to be a transcriptional regulator involved in the stress response
AreB (AFUB_029020)	GATA zinc finger transcription factor, leucine zipper motif, negative regulator of nitrogen catabolism
FkhA (AFUB_037220)	Forkhead family transcription factor; rate-limiting activator of replication origins; evolutionarily conserved regulator of lifespan; major role in expression of G_2_/M-phase genes

10.1128/mBio.02962-19.1 FIG S1 Null kinase mutants grown under different stress conditions. A. fumigatus conidia (1 × 10^5^) were inoculated on solid minimal medium (MM) with different concentrations of Congo red, calcofluor white (CFW), sorbitol, and caspofungin and grown for 5 days at 37°C. Download FIG S1, PDF file, 0.1 MB. Copyright © 2020 Mattos et al. 2020 Mattos et al. This content is distributed under the terms of the Creative Commons Attribution 4.0 International license.

None of the protein kinase null mutants lost the caspofungin paradoxical effect (CPE; the escape of A. fumigatus from caspofungin inhibition at concentrations above the MIC) (3). In contrast, three TF null mutants corresponding to AFUB_009970, AFUB_019790, and AFUB_037220 showed a significantly decreased CPE. Therefore, this phosphoproteomic study has facilitated the identification of novel protein kinases and TFs involved in osmotic, cell wall, and caspofungin stress responses.

### A. fumigatus HOG MAPKs are important for the phosphoproteomic response to cell wall damage.

We previously demonstrated that the MpkC and SakA kinases of the HOG pathway play a role in the response to osmotic, oxidative, and cell wall stresses, in addition to influencing CWI MpkA signaling (29). To assess the involvement of MpkC and SakA in the modulation of phosphorylation cascades activated by cell wall damage, the phosphoproteome of the Δ*sakA*, Δ*mpkC*, and double Δ*sakA* Δ*mpkC* mutants was examined in the absence (T0) or presence of CR for 30 min (CR30).

**FIG 4:**
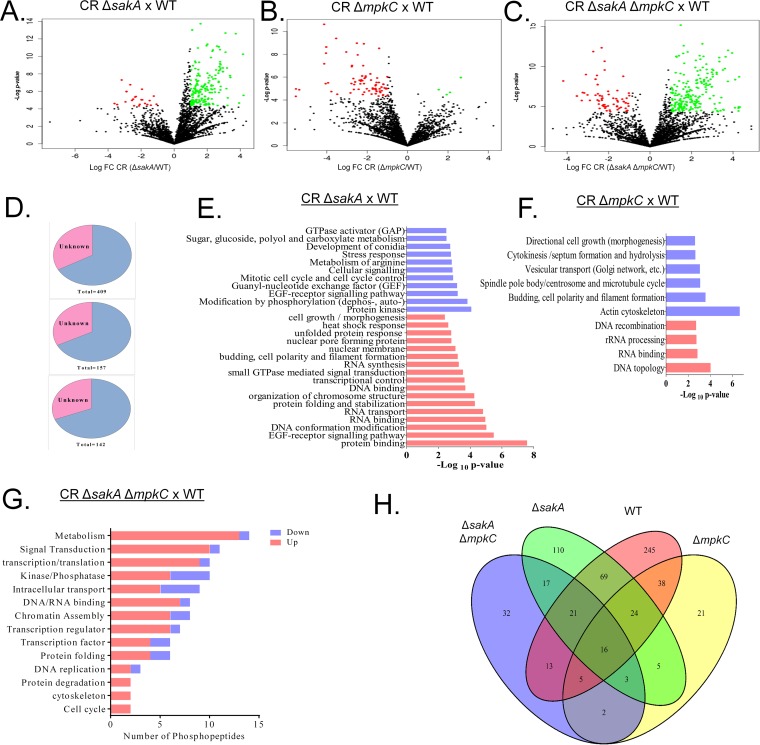
Phosphorylation profile of proteins from A. fumigatus and MAPK mutants during incubation with Congo red (CR 10 min). (A to C) Volcano plots of total phosphopeptides identified in *ΔsakA* mutant versus wild type (WT), *ΔmpkC* mutant versus WT, and the double *ΔsakA ΔmpkC* mutant versus WT, respectively. (D) Percentage of proteins with unknown and known function in all three phosphoproteomes *ΔsakA* (top); *ΔmkpC* (middle); and *ΔsakA ΔmpkC* (bottom). All of them showed more than 60% characterized proteins. (E to G) Functional categorization of approximately 75% of proteins with known function differentially phosphorylated in *ΔsakA*, *ΔmpkC*, and *ΔsakA ΔmpkC* strains during CR stress. (H) Venn diagram of total differentially phosphorylated proteins (*n* = 621) representing the number of common phosphoproteins between strains in each intersection (each strain has a set of differentially phosphorylated proteins after CR treatment compared to its untreated control). Thus, the WT strain has a total set of 431 phosphoproteins from which a unique set of 245 is not shared with any other condition, 69 are shared only with the *ΔsakA* mutant, 13 are shared only with the *ΔsakA ΔmpkC* mutant, and 38 are shared only with the *ΔmpkC* mutant, while 16 phosphoproteins are shared simultaneously by the four strains.

When the CR30-treated Δ*sakA* mutant was compared to CR30-treated WT, a total of 4,835 phosphopeptides were identified (location probability >75% [Table S2A]); 409 phosphopeptides corresponding to 268 proteins show significant differences between mutant and WT (Student *t* test with *P* < 0.05) (Fig. 4A). A prevalence of phosphorylation events was observed (67 phosphopeptides with total phosphorylation exclusive for the mutant strain and 284 with increased phosphorylation), with only 33 phosphopeptides with total dephosphorylation (exclusive to WT) and 25 with decrease of phosphorylation in the mutant strain (Fig. 4A and Table S2B). For the same comparison without CR treatment (T0), a total of 3,741 phosphopeptides were selected (location probability >75%) with only 27 phosphopeptides significantly different between *ΔsakA* and WT strains (*P* < 0.05) (Table S3A and B).

10.1128/mBio.02962-19.4 TABLE S2 (A) Complete list of phosphosites identified comparing the Δ*sakA* null mutant with the wild-type strain in the presence of Congo red. (B) List of phosphosites identified comparing the Δ*sakA* null mutant with the wild-type strain in the presence of Congo red with *P* < 0.05. Download Table S2, XLSX file, 2.7 MB. Copyright © 2020 Mattos et al. 2020 Mattos et al. This content is distributed under the terms of the Creative Commons Attribution 4.0 International license.

10.1128/mBio.02962-19.5 TABLE S3 (A) Complete list of phosphosites identified comparing the Δ*sakA* null mutant with the wild-type strain. (B) List of phosphosites identified comparing the Δ*sakA* null mutant with the wild-type strain with *P* < 0.05. Download Table S3, XLSX file, 2.0 MB. Copyright © 2020 Mattos et al. 2020 Mattos et al. This content is distributed under the terms of the Creative Commons Attribution 4.0 International license.

For the comparison between *ΔmpkC* and WT strains after 30-min CR incubation, 4,638 phosphopeptides were identified (location probability >75% [Table S4A]); 157 phosphopeptides corresponding to 118 proteins were observed with significant differences between mutant and WT (Student *t* test with *P* < 0.05) (Fig. 4A and Table S4B). The phosphorylation profile of the Δ*mpkC* strain was the opposite of Δ*sakA* strain, with a major reduction of phosphorylation (37 phosphopeptides with total dephosphorylation, 94 with decreased phosphorylation), and 6 phosphopeptides with increased phosphorylation and 20 with total phosphorylation (Table S4B). For the comparison between the controls (T0), a total of 3,675 phosphopeptides were identified (location probability >75%), with 157 phosphopeptides significantly differing between *ΔmpkC* and WT strains (*P* < 0.05) (Table S5A and B).

10.1128/mBio.02962-19.6 TABLE S4 (A) Complete list of phosphosites identified comparing the Δ*mpkC* null mutant with the wild-type strain in the presence of Congo red. (B) List of phosphosites identified comparing the Δ*mpkC* null mutant with the wild-type strain in the presence of Congo red with *P* < 0.05. Download Table S4, XLSX file, 2.6 MB. Copyright © 2020 Mattos et al. 2020 Mattos et al. This content is distributed under the terms of the Creative Commons Attribution 4.0 International license.

10.1128/mBio.02962-19.7 TABLE S5 (A) Complete list of phosphosites identified comparing the Δ*mpkC* null mutant with the wild-type strain. (B) List of phosphosites identified comparing the Δ*mpkC* null mutant with the wild-type strain with *P* < 0.05. Download Table S5, XLSX file, 2.2 MB. Copyright © 2020 Mattos et al. 2020 Mattos et al. This content is distributed under the terms of the Creative Commons Attribution 4.0 International license.

In the *ΔmpkC ΔsakA* response to CR for 30 min, 4,855 phosphopeptides were identified (localization probability >75%), and 142 phosphopeptides corresponding to 111 proteins have significant phosphorylation change between mutant and WT (Fig. 4C and Table S6A and B). The double mutant also showed a profile of increased phosphorylation, as observed for the WT and *ΔsakA* strains in the CR 30-min response, with 26 phosphopeptides with total dephosphorylation, 12 with decreased phosphorylation, 70 with increased phosphorylation, and 34 with total phosphorylation (Fig. 4C). For the comparison without CR (T0), 3,741 phosphopeptides were observed, with only 54 significantly different between mutant and WT (Table S7A and B).

10.1128/mBio.02962-19.8 TABLE S6 (A) Complete list of phosphosites identified comparing the double mutant with the wild-type strain in the presence of Congo red. (B) List of phosphosites identified comparing the double mutant with the wild-type strain in the presence of Congo red with *P* < 0.05. Download Table S6, XLSX file, 2.7 MB. Copyright © 2020 Mattos et al. 2020 Mattos et al. This content is distributed under the terms of the Creative Commons Attribution 4.0 International license.

10.1128/mBio.02962-19.9 TABLE S7 (A) Complete list of phosphosites identified comparing the double mutant with the wild-type strain. (B) List of phosphosites identified comparing the double mutant with the wild-type strain with *P* < 0.05. Download Table S7, XLSX file, 2.1 MB. Copyright © 2020 Mattos et al. 2020 Mattos et al. This content is distributed under the terms of the Creative Commons Attribution 4.0 International license.

To determine Gene Ontology (GO) categories overrepresented in the mutant phosphoproteomes, we carried out a functional categorization by FungiFun analysis (Fig. 4D to F). For all mutants, at least 65% of the phosphopeptides have known function and less than 35% are hypothetical proteins (Fig. 4D). For *ΔsakA* mutants, most of the proteins with increased phosphorylation upon CR exposure are related to protein binding, epidermal growth factor (EGF) receptor signaling pathway, DNA conformation modification, and DNA or RNA binding. For the group of proteins with decreased phosphorylation upon CR exposure, most of them are protein kinases and proteins related to modification by phosphorylation (phosphorylation, dephosphorylation, or autophosphorylation) (Fig. 4E). For the *ΔmpkC* mutant, proteins with increased phosphorylation are mainly related to RNA topology, RNA binding, and rRNA processing, while proteins with decreased phosphorylation are related to cytoskeleton, such as actin cytoskeleton; budding, cell polarity, and filament formation; and spindle pole body/centrosome and microtubule cycle (Fig. 4F). The functional analysis of the double mutant phosphoproteome in CR response by FungiFun did not show enrichment of any pathway (data not shown). However, by using GO enrichment, we were able to identify phosphorylation of proteins related to metabolism, signal transduction, transcription/translation, cell cycle, cytoskeleton, and protein degradation. Enrichment for protein kinases/phosphatases and proteins related to intracellular transport, for example, has been identified in the double mutant with both increased and decreased phosphorylation (Fig. 4G).

A Venn diagram was generated for correlating the phosphorylation profile between each strain treated with CR (Fig. 4H). Although each strain had a unique set of phosphoproteins (245 for WT, 110 for Δ*sakA* mutant, 21 for Δ*mpkC* mutant, and 32 for Δ*sakA* Δ*mpkC* mutant), among the three mutant strains the Δ*sakA* strain showed the highest similarity to the wild-type phosphoproteome during CR exposure, with 69 shared phosphoproteins. The low number of common phosphoproteins among all conditions (16 phosphoproteins) in addition to the low number of common phosphoproteins between the Δ*mpkC* and the double Δ*sakA* Δ*mpkC* mutants (only 2 phosphoproteins) may indicate their independent roles in distinct pathways involved in the CR response.

### Interaction networks of identified A. fumigatus phosphoproteins.

**FIG 5:**
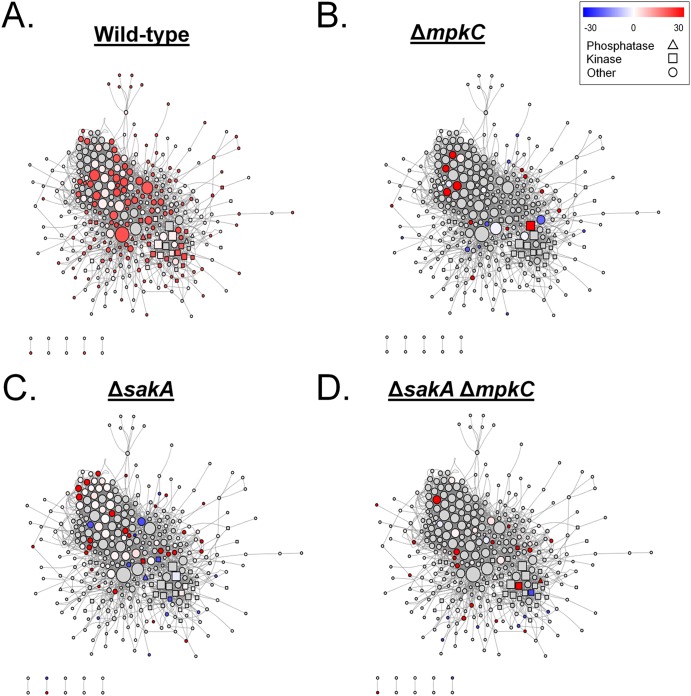
General A. fumigatus functional protein association network based on the protein phosphorylation profile during incubation with Congo red (CR 10 min). The whole set of differentially phosphorylated proteins from the wild-type (WT), Δ*sakA*, Δ*mpkC*, and Δ*sakA* Δ*mpkC* strains during CR stress was combined for the generation of a general protein association network. Each node represents a protein that is differentially phosphorylated in at least one strain under CR stress. Node colors are divided into gray nodes, for proteins which were not differentially phosphorylated, and heatmap-colored nodes (blue-white-red), for proteins which were differentially phosphorylated in each strain in relation to its control (the same strain without any stress condition). Node shapes represent molecular functions and are divided into triangles (phosphatases), squares (kinases), and circles (other functions). Each edge represents a functional protein association retrieved from the STRING server (medium confidence threshold of 0.4 for the interaction score), and node sizes represent the degree of each node (number of edges connected to the node). It is important to note that not all the differentially phosphorylated proteins are present in the network, as many proteins did not present any functional associations within the whole set. Changes in the color patterns of nodes from panels A to D represent changes in the differential phosphorylation of the proteins comprising the network in WT, Δ*sakA*, Δ*mpkC*, and Δ*sakA* Δ*mpkC* strains, respectively, during CR stress.

Pairwise analyses identified the differentially phosphorylated proteins after exposure to CR in the different strains. This showed that the phosphoproteomes of wild-type and Δ*sakA* strains were functionally enriched for serine/threonine protein kinase domains and RNA recognition motif domains, respectively (see Table S1E for less representative functional enrichments). However, the phosphoproteomes for the Δ*mpkC* and the double mutant Δ*sakA* Δ*mpkC* strains did not have any functional enrichment. The individual sets of differentially phosphorylated proteins in wild-type, Δ*sakA*, Δ*mpkC*, and the double mutant Δ*sakA* Δ*mpkC* strains were combined, yielding 595 proteins which were used to generate a single functional protein association network. The network was composed of 336 nodes and 1862 interactions between nodes (Fig. 5), revealing the enrichment for protein kinases, RNA recognition motif domains, and MAPK signaling pathways (Table S1E). The change of the color pattern in the nodes of Fig. 5A to D highlights the overall network rewiring in terms of differentially phosphorylated proteins for each strain (WT and Δ*sakA*, Δ*mpkC*, and Δ*sakA* Δ*mpkC* mutants) in the presence of CR compared to their respective controls (untreated).

**FIG 6:**
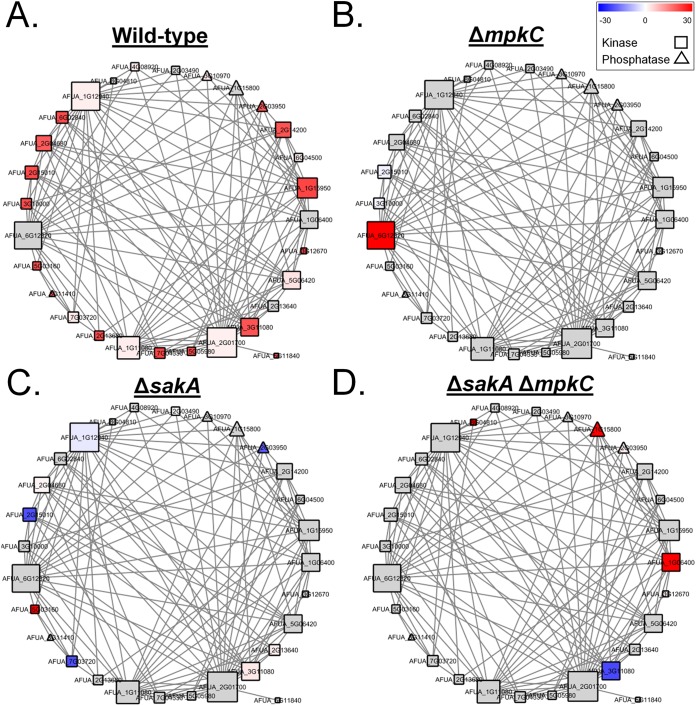
Subnetwork of functional protein associations based only on the phosphorylation profile of protein kinases and phosphatases during incubation with Congo red (CR 10 min). The subset of differentially phosphorylated kinases and phosphatases from the wild-type (WT), Δ*sakA*, Δ*mpkC*, and Δ*sakA* Δ*mpkC* strains during CR stress was extracted from the general protein association network. Each node represents a protein that was differentially phosphorylated in at least one strain under CR stress. Node colors are divided into gray nodes (proteins that were not differentially phosphorylated) and heatmap-colored nodes (blue-white-red; proteins which were differentially phosphorylated in each strain in relation to its untreated control). Node shapes represent molecular functions and are divided into squares (kinases) and triangles (phosphatases). Each edge represents a functional protein association retrieved from the STRING server (medium confidence threshold of 0.4 for the interaction score). Node size is representative of the number of edges connected to the node. Note that not all the differentially phosphorylated proteins are present in the network, as for many proteins no functional associations were available. Changes in the color patterns of nodes from panels A to D represent changes in the differential phosphorylation of the proteins comprising the network in WT, Δ*sakA*, Δ*mpkC*, and Δ*sakA* Δ*mpkC* strains, respectively, during CR stress.

Protein kinases and phosphatases play essential roles in eukaryotic signal transduction pathways. The network analysis showed enrichment for protein kinase and phosphatase domains; hence, a subnetwork highlighting only differentially phosphorylated kinases and phosphatases was extracted (Fig. 6 and Table S1E). The phosphorylation profiles among strains after exposure to CR changed dramatically. In the wild-type strain (Fig. 6A), most of the kinases and phosphatases in the network showed increased phosphorylation compared to the untreated control, including all of the highly connected kinases except for the mitogen-activated protein kinase MpkB (AFUA_6G12820). There was an overall decrease of differentially phosphorylated kinases and phosphatases in Δ*mpkC*, Δ*sakA*, and Δ*sakA* Δ*mpkC* strains in comparison to the wild-type CR response (Fig. 6B to D). In the Δ*mpkC* strain, MpkB was the only kinase with increased phosphorylation (Fig. 6B). In contrast to the other strains, the Δ*sakA* mutant presented a higher abundance of kinases and phosphatases with decreased phosphorylation after CR exposure (Fig. 6C). The double Δ*sakA* Δ*mpkC* mutant presented a specific set of differentially phosphorylated proteins, which were not modulated in the same manner in any of the other networks (Fig. 6D). This included the increased phosphorylation of the serine/threonine protein kinase SchA (AFUA_1G06400) and protein phosphatase 2C PtcA (AFUA_1G15800), plus the decreased phosphorylation of the MAP kinase kinase kinase BckA (AFUA_3G11080).

### Phosphosite validation.

**FIG 7:**
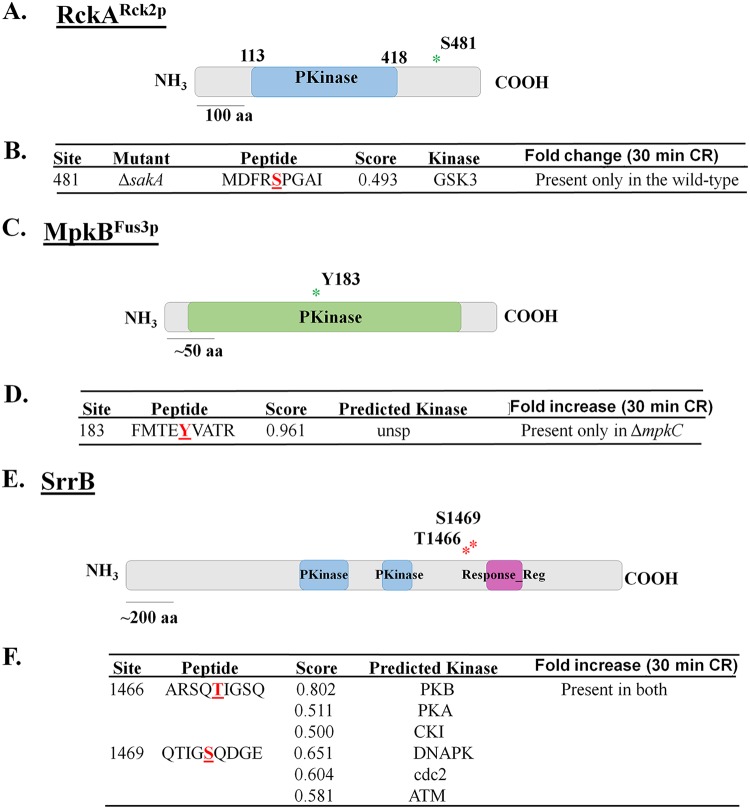
Sites of phosphorylation (S or T) identified during response to Congo red (CR) in wild-type or three kinase null mutant strains. (A, C, and E) Schematics of the kinases showing their kinase domain (blue or green boxes) and position of phosphorylated amino acid residues highlighted in red. (B, D, and F) Phosphorylation sites identified in the phosphoproteome, according to the conditions indicated. For each residue, a score for a kinase site as predicted by Group-based Prediction System 3.0 (GPS; with medium-stringency cutoff) is indicated.

Two approaches were adopted to determine the effects of the identified protein phosphorylation events on A. fumigatus CWI: (i) the solved protein structures of available homologues deposited in the Protein Data Bank (PDB) were assessed and (ii) serine/tyrosine residues were mutated to alanine/phenylalanine, or to phosphomimetic aspartate and functionality was assessed. These phosphomutations were introduced by using a CRISPR-Cas9 system. Only a single three-dimensional (3D) structure was available for RckA^Rck2p^, since no solved structures for the phosphorylated regions of the other chosen proteins were deposited in the PDB. We concentrated our attention on a few proteins, aiming to validate the results obtained by the phosphoproteomics analysis (Fig. 7). Therefore, RckA, MpkB, and SrrB were selected for functional characterization to validate the phosphoproteomics observations.

**FIG 8:**
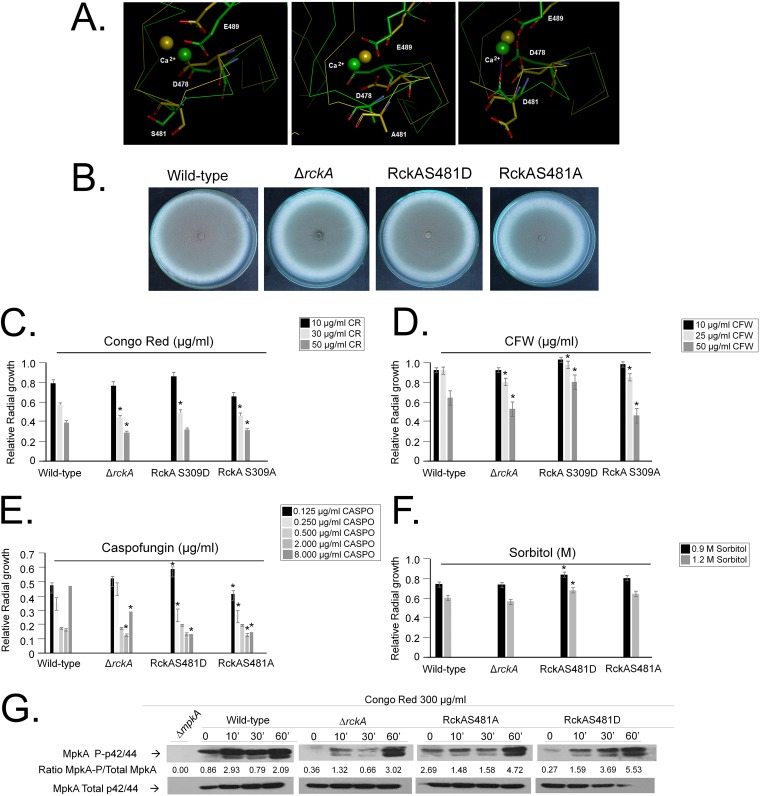
Phosphomutants for RckA serine phosphorylation sites. (A) Molecular modeling for RckA wild type and predicted phosphomutants. (B to F) Growth phenotypes of the wild type and Δ*rckA*, RckA^S481D^, and RckA^S481A^ mutants grown for 5 days at 37°C on minimal medium (MM) or MM plus cell wall-damaging or osmotic stress agents (Congo red [CR], calcofluor white [CFW], caspofungin, and sorbitol). (G) Western blot analysis for MpkA phosphorylation in the wild type and Δ*rckA*, RckA^S481D^, and RckA^S481A^ mutants grown for 16 h in MM and transferred to MM plus 300 μg/ml of Congo red. Signal intensities were quantified using the Image J software by dividing the intensity of phosphorylated MpkA (MpkA∼P) by MpkA.

RckA^Rck2p^ (AFUA_2G03490) is the homologue of the S. cerevisiae Rck2p calcium/calmodulin-dependent protein kinase involved in response to oxidative and osmotic stresses. A single phosphorylation site was identified in RckA (477-MDFR**S**PGAI-485), which was phosphorylated only when the wild type was exposed to CR (Fig. 7A and B). A BLASTP search performed for *RCK1* homologues with solved structures deposited in the PDB identified a calcium/calmodulin-dependent serine/threonine kinase from Cryptosporidium parvum. The crystal structure of full-length CpCDPK3 (cgd5_820; PDB accession no. 3LIJ) protein in complex with Ca^2+^ and AMP-PNP (adenylyl-imidodiphosphate), at 1.9-Å resolution, was used as a template to build three homology models for RckA with respect to the wild-type S481 residue (corresponding to S309 in C. parvum) and the two phosphomutations on the S481 residue (S481A and S481D). The pairwise alignments used for homology modeling were refined by structural inspection to avoid gaps (by deletion or insertion) inside secondary structure elements. Models were analyzed to reveal satisfactory atomic contacts, chemical environment, and stereochemistry as well as high-resolution structures (above 2.0 Å), and they indicate that residue S481 is inserted at the middle of the short loop of the calcium-binding EF-hand motif (Fig. 8A).

The three RckA models with different S481 residues were used in long-trajectory molecular dynamics simulations, to assess differences in structural stability and conformation. RckA models containing S481 and S481A were more stable, while the highest main chain conformational change was observed in the S481D model, whose aspartic residue moves from a buried to a solvent-accessible conformation, thus providing an additional calcium-binding residue. This protein shows different patterns of calcium binding and protein activity from the other two (Fig. 8A).

The Δ*rckA*, RckA^S481D^, and RckA^S481A^ strains have growth rates and conidiation comparable to the wild-type strain (Fig. 8B). The Δ*rckA* and RckA^S481A^ strains were more sensitive to CR- and CFW-induced cell wall damage, while the RckA^S481D^ strain was more resistant to CFW (Fig. 8C and D). Surprisingly, all three RckA mutant strains were more sensitive to caspofungin and completely or partially lost the CPE (Fig. 8E). There were no differences in osmotic stress tolerance among the wild-type, Δ*rckA*, and RckA^S481A^ strains. However, the RckA^S481D^ strain was more resistant to high sorbitol concentrations than the wild-type or other mutant strains (Fig. 8F). MpkA phosphorylation in the Δ*rckA* mutant upon exposure to CR was similar to the wild-type strain (Fig. 8G). The RckA^S481A^ mutant had increased MpkA phosphorylation levels that decreased after exposure to CR for 10 and 30 min but increased about twice at 60 min of exposure (Fig. 8G). The RckA^S481D^ mutant has higher levels of MpkA phosphorylation than the wild-type strain upon exposure to CR (Fig. 8G). Taken together, the data suggest that *rckA* mutants impact MpkA phosphorylation upon cell wall damage, strongly suggesting that RckA is important for the CWI pathway.

**FIG 9:**
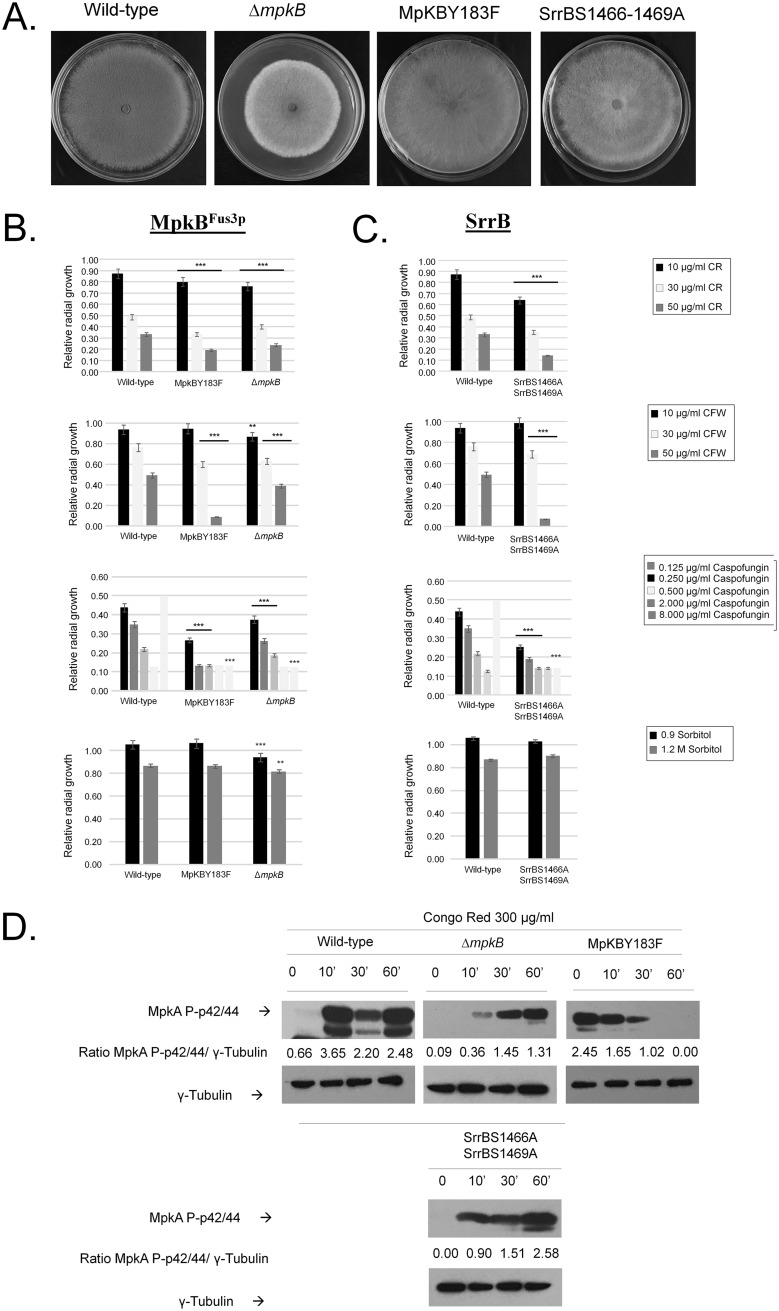
Phosphomutants for MpkB and SrrB serine phosphorylation sites. (A to C) Phenotypes of the wild type and Δ*mpkB*, MpkB^Y183F^, and SrrB^S1466–1469A^ mutants grown for 5 days at 37°C on minimal medium (MM) or MM plus cell wall-damaging or osmotic stress agents (Congo red [CR], calcofluor white [CFW], caspofungin, and sorbitol). (D) Western blot analysis for MpkA phosphorylation in wild type and Δ*mpkB*, MpkB^Y183F^, and SrrB^S1466–1469A^ mutants grown for 16 h in MM and transferred to MM plus 300 μg/ml of Congo red. Signal intensities were quantified using the Image J software by dividing the intensity of MpkA∼P by γ-tubulin.

MpkB (AFUA_6G12820) is the homologue of S. cerevisiae Fus3p mitogen-activated serine/threonine protein kinase, involved in mating. A single phosphorylation site was identified in MpkB (179-FMTE**Y**VATR-187) that was phosphorylated only when Δ*mpkB* was exposed to CR (Fig. 7C and D). The Δ*mpkB* strain has reduced growth and conidiation (28), while the MpkB^Y481F^ mutant has similar growth rates and conidiation as the wild-type strain (Fig. 9A). The Δ*mpkB* and MpkB^Y183F^ strains were more sensitive to CR, CFW, caspofungin (also exhibiting reduction in the CPE), and osmotic stress (Fig. 9B). MpkA phosphorylation in the Δ*mpkB* mutant was 10-fold less than for the wild type after 10-min exposure to CR (Fig. 9D), while in the MpkB^Y183F^ mutant MpkA phosphorylation was 4-fold higher in untreated cells, but 2-fold lower after 10-min exposure to CR, than in the wild-type strain (Fig. 9D). After many attempts, we were not able to construct an MpkB^Y183D^ mutant.

SrrB (AFUA_2G15010) is a putative response regulator of a two-component phosphorelay system. Two phosphorylation sites (1462-ARSQ**T**IG**S**QGE-1472) were identified as phosphorylated in both wild-type and Δ*sakA* strains exposed to CR (Fig. 7E and F). The SrrB^S1466–1469A^ mutant had comparable growth rates and conidiation as the wild-type strain (Fig. 9A). The SrrB^S1466–1469A^ strain was more sensitive to CR, CFW, and caspofungin (with a reduction in the CPE) and had growth comparable to the wild-type strain under high-osmotic-stress conditions (Fig. 9C). MpkA phosphorylation in the SrrB^S1466–1469A^ mutant upon exposure to CR for 10 min was 3-fold less than in the wild-type strain (Fig. 9D).

Taken together, these results suggest that the RckA^S481A^ mutation mimics the loss-of-function mutation, while the RckA^S481D^ mutation allows the mutant strain to be more efficient in coping with cell wall and osmotic stress conditions. However, for all the other phosphomutants there was an increase in the sensitivity to cell wall-damaging agents and a reduction in the MpkA phosphorylation upon CR stress, suggesting these phosphosites could be important for the MpkA modulation and CWI pathway regulation.

## DISCUSSION

The filamentous fungal cell wall is essential for survival and serves as a primary means by which fungi interact with their environment. However, relatively limited information is available regarding the major regulatory events involved in cell wall biosynthesis and repair. The A. fumigatus module PkcA-MpkA-RlmA is essential for the CWI (36, 41–45). To learn more about the main regulatory posttranslational events that occur during CWI activation, we have investigated the global phosphoproteomics of A. fumigatus during cell wall damage caused by CR. A total of 756 high-confidence phosphorylation sites were identified in the A. fumigatus wild-type strain. A total of 380 were exclusive to CR-treated samples, and 3 were observed only in the control, while 373 were observed in the presence and absence of CR. These phosphopeptides correspond to 485 proteins. According to FunCat annotation enrichment and the interaction network analyses of this novel phosphoproteome, the proteins phosphorylated in response to CR were involved in a variety of important biological processes, including signal transduction (osmosensing and response, Ca^2+^-mediated signal transduction, and MAPKKK cascade), stress response, protein kinases, actin cytoskeleton, and budding cell polarity and filament formation. These results emphasize the importance of osmotic stress, calcium metabolism, and MAPKs in the A. fumigatus CWI pathway, as previously demonstrated (29, 34, 78–82).

We also investigated the role played by the A. fumigatus HOG MAPKs MpkC and SakA. These two MAPKs are important for the MpkA phosphorylation during cell wall damage (29). Recently, SakA was demonstrated to physically associate with MpkC upon osmotic and CR-induced cell wall stresses (34). We were not able to identify a phosphorylation pattern that explains how MpkC modulates SakA function. Under cell wall stress, SakA also physically interacts with the CWI MAPK, MpkA, and the PtcB MAPK phosphatase, suggesting a close connection between the HOG and CWI pathways. Cross-talk between the HOG and CWI pathways has been observed in several fungal species, including S. cerevisiae, Candida albicans, Fusarium graminearum, and Pyrenophora graminea (56–64). Now, we show that the absence of these two HOG pathway-associated MAPKs, MpkC and SakA, abolishes the remodeling of the phosphoproteome seen in the wild-type fungus in response to cell wall damage, and we also reveal novel proteins potentially involved in cell wall stress response.

**FIG 10:**
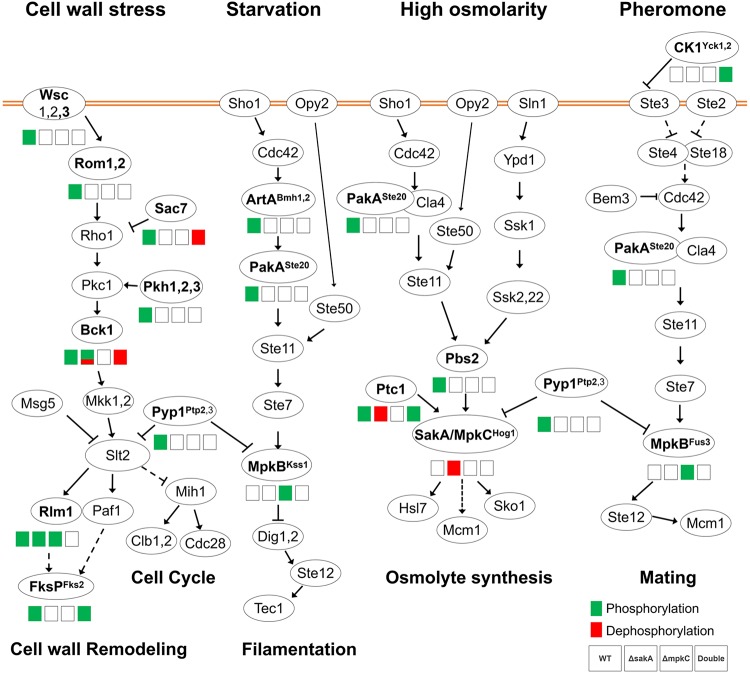
Differential phosphorylation of MAPK pathways during response to cell wall damage. The wild-type, Δ*sakA*, Δ*mpkC*, and Δ*sakA* Δ*mpkC* strains were exposed to CR for 30 min. Phosphorylated (green boxes) or dephosphorylated (red boxes) proteins in the presence of CR are shown in the different MAPK pathways. Superscript names correspond to the respective homologue genes in S. cerevisiae. Proteins with no phosphorylation modulation are represented with the S. cerevisiae corresponding name, adapted from the MAPK signaling pathway, yeast, Aspergillus fumigatus, in KEGG (Kyoto Encyclopedia of Genes and Genomes [https://www.genome.jp/kegg/]).

Our global phosphoproteome network analysis showed an enrichment for protein kinases, RNA recognition motif domains, and the MAPK signaling pathway. In contrast to the wild-type strain, there was an overall decrease of differentially phosphorylated kinases and phosphatases, among them RckA^Rck2p^, MpkB^Fus3p^, and SrrB, in Δ*mpkC*, Δ*sakA*, and Δ*sakA* Δ*mpkC* strains. We introduced phosphomutations in these proteins and assessed their tolerance of cell wall-damaging agents and osmotic stress conditions. Although it is very difficult to assign a single role for a specific protein kinase or phosphatase in the CWI pathway, the validation of our results by introducing corresponding phosphomutations in two protein kinases (RckA and MpkB) and a putative response regulator of a two-component phosphorelay system (SrrB) strongly indicated the importance of the osmotic stress and the cross-talk among HOG and MpkB^Fus3p^ pathways in the CWI pathway. This is reflected in the fact that most of the A. fumigatus putative homologues of the components of the three MAPK pathways were differentially phosphorylated or dephosphorylated in the presence or absence of cell wall damage (Fig. 10).

We discovered several protein kinases and TFs involved and validated their importance in the A. fumigatus cell wall damage response. None of the 11 kinase null mutants were found to be more sensitive or resistant to several cell wall or osmotic stresses. Hence, further experimental validation will help to determine which kinases are important to the osmotic stress and cell wall damage response pathways. In contrast, all five TF null mutants showed cell wall stress-related phenotypes. This included the AreB (AFUB_029020) GATA zinc finger transcription factor, which negatively regulates nitrogen catabolism (65). The A. nidulans *areB* null mutant has significant phenotypic effects on the utilization of specific carbon sources, confirming its role in the regulation of carbon metabolism (66). Considering the importance of carbon source assimilation in the remodeling of the cell wall could explain why in A. fumigatus AreB is important for the cell wall damage response. Another TF was AsgA (AFUB_019790), the homologue of S. cerevisiae Asg1p, a zinc cluster protein proposed to be a transcriptional regulator involved in the stress response. The S. cerevisiae Δ*asg1* mutant has a respiratory defect and is sensitive to CFW (67). It is possible that A. fumigatus AsgA is similarly involved in some aspects of the cell wall damage response. In S. cerevisiae, the forkhead family transcription factor Fkh2 is involved in the activation of early-firing replication (68) and Fkh2 mutations suppress the checkpoint-defective phenotype of both HOG and cell wall integrity MAPK mutations (63). Therefore, the A. fumigatus homologue, FkhA, could be a putative CWI checkpoint for cell cycle progression in A. fumigatus.

Our global phosphoproteome analysis of the fungal response to cell wall damage has provided the identification of several novel proteins that are important for cell wall stress tolerance. These results will provide opportunities to understand how the signal transduction networks that modulate the integrity of the cell wall function. In addition, our results highlight potential new targets for interference in fungal signaling events and cell wall remodeling to promote the efficacy of existing antifungals while impeding virulence.

## MATERIALS AND METHODS

### Strains and growth medium.

Aspergillus fumigatus strain CEA17 was used for CRISPR-Cas9 editing (Table 2). Fungal strains were cultivated in liquid YPD medium (1% yeast extract, 1% polypeptone, and 1% glucose) or in solid minimal medium (MM): 1% glucose, nitrate salt solution (1×), trace metal solution (1×), 0.001% thiamine, and 2% agar (69, 70). For transformation, TM medium (transformation medium; with 1 M sucrose instead of 1% glucose) was used. After the confirmation of the mutations by DNA sequencing, fungal strains were grown in MM supplemented with 10 mM uridine (Uri) and 10 mM uracil (Ura) and 5-fluoroorotic acid (5-FOA) (71). Escherichia coli DH5α was used for amplification of CRISPR vectors and was grown in solid or liquid LB medium containing 100 μg/ml ampicillin.

**TABLE 2:** Strains used in this work

Name	Genotype	Source or reference
CEA17	Wild-type strain	FGSC A1159
*ΔsakA*	Δ*sakA*::*hph*	29
*ΔmpkC*	Δ*mpkC*::*ptrA*	29
*ΔsakA* Δ*mpkC*	Δ*sakA*::*hph* Δ*mpkC*::*ptrA*	29
*ΔmpkB*	*ΔmpkB*::*ptrA*	28
MFIG001	Δ*akuB pyrG*^+^	FGSC A1160
Δ*hriA* (AFUB_032300)	Δ*hriA*::*hph* Δ*akuB*	This study
Δ*srrB* (AFUB_030660)	Δ*srrB*::*hph* Δ*akuB*	This study
Δ*kin1* (AFUB_010510)	Δ*kin1*::*hph* Δ*akuB*	This study
Δ*snfA* (AFUB_018770)	Δ*snfA*::*hph* Δ*akuB*	This study
Δ*cmkA* (AFUB_029320)	Δ*cmkA*::*hph* Δ*akuB*	This study
Δ*prr1* (AFUB_029820)	Δ*prr1*::*hph* Δ*akuB*	This study
Δ*pkhA* (AFUB_036500)	Δ*pkhA*::*hph* Δ*akuB*	This study
Δ*cmkC* (AFUB_053520)	Δ*cmkC*::*hph* Δ*akuB*	This study
Δ*kfsA* (AFUB_014350)	Δ*kfsA*::*hph* Δ*akuB*	This study
Δ*oca2* (AFUB_059390)	Δ*oca2*::*hph* Δ*akuB*	This study
Δ*fpkA* (AFUB_059090)	Δ*fpkA*::*hph* Δ*akuB*	This study
ΔAFUB_004210	ΔAFUB_004210 Δ*akuB*	This study
ΔAFUB_009270	ΔAFUB_009270 Δ*akuB*	This study
Δ*asgA* (AFUB_019790)	Δ*asgA*::*hph* Δ*akuB*	This study
Δ*areB* (AFUB_029020)	Δ*areB*::*hph* Δ*akuB*	This study
Δ*fkhA* (AFUB_037220)	Δ*fkhA*::*hph* Δ*akuB*	This study
Afu6g12820_**mpkB**_184Phe	Y184P mutation	This study
Afu2g15010_**srrB**_1466Ala1469Ala	S1466A and S1469A mutations	This study
Afu2g15010_**srrB**_1466Asp1469Asp	S1466D and S1469D mutations	This study

### Fungal growth, protein extraction, digestion, and phosphoenrichment.

A. fumigatus conidia (1 × 10^7^) from the wild-type (WT) and mutant strains (*ΔsakA*, *ΔmpkC*, and *ΔsakA ΔmpkC* strains [Table 2]) (72) were inoculated in YG medium {0.5% yeast extract, 1% dextrose; 0.1% trace elements [22.0 g/liter ZnSO_4_, 11 g/liter boric acid, 5 g/liter MnCl_2_, 5 g/liter FeSO_4_, 1.6 g/liter CoCl_2_, 1.6 g/liter CuSO_4_, 1.1 g/liter (NH4)_2_MoO_4_]} and incubated for 16 h under 200-rpm rotation and 37°C. After that, 300 μg/ml of Congo red (CR) was added, followed by incubation for 10 min (or time zero, as control [T0]). Mycelia were then filtered using a vacuum system and frozen by liquid nitrogen. Frozen mycelia were macerated, and protein was extracted by the addition of TNE buffer (50 mM Tris-HCl [pH 7.5], 140 mM NaCl, 5 mM EDTA, phosphatase inhibitor cocktail [PhosphoStop; Roche], EDTA-free protease inhibitor cocktail [Roche], and 0.1 mM phenylmethylsulfonyl fluoride [PMSF]) and incubation for 15 min under agitation following centrifugation at 10,000 × *g* for 20 min. The supernatant was collected, and total protein was quantified by the Bradford assay. Five hundred micrograms of protein was purified by 3-kDa-molecular-weight-cutoff ultrafiltration (Centriprep YM-3; Millipore, Billerica, MA) followed by trichloroacetic acid (TCA) precipitation. TCA was added to protein extract at a 10% (wt/vol) final concentration, vortexed for 15 s, and placed on ice for a minimum of 30 min. Sample were centrifuged at 14,000 × *g* for 15 min, and the supernatant was discarded. The pellet was washed three times with cold acetone with centrifugation at 14,000 × *g*, 4°C, for 10 min. Protein pellets were reduced by the addition of dithiothreitol (DTT) and alkylated by the addition of iodoacetamide. Protein digestion was performed by the addition of sequencing-grade trypsin (Promega) (1:50) for 16 h at 37°C. Phosphopeptides were enriched with the Pierce TiO_2_ phosphopeptide enrichment and cleanup kit (Thermo Fisher Scientific) according to the manufacturer’s instructions. The strains used in this paper are listed in Table 2.

### LC-MS/MS.

Phosphopeptide identification and analysis were performed with at least 4 replicates on a nano-liquid chromatograph (nanoLC) (NanoAcquity; Waters Corporation, Milford, MA) coupled to an Orbitrap Fusion Tribrid mass spectrometer (Thermo Fisher, San Jose, CA). Samples were resolved in 10 μl of acetonitrile (ACN)-H_2_O-formic acid (FA) (5/95/0.1, vol/vol/vol) for LC-MS/MS analysis. For each run, 2 μl of sample was loaded on a 100-μm (inner diameter [i.d.]) by 20-mm, 5-μm C_18_ precolumn at a flow rate of 4 μl/min for 5 min, using a loading buffer of ACN-H_2_O-FA (5/95/0.1, vol/vol/vol). Peptide separation was performed on a 75-μm (i.d.) by 180-mm, 5-μm C_18_ analytical column at a flow rate of 250 nl/min in a 95-min gradient by using mobile phase A of 0.1% formic acid in water and mobile phase B of 0.1% FA in acetonitrile. The gradient elution started at 5% mobile phase B, was increased to 35% at 60 min and 80% at 65 min, and was held at 80% for 5 min before a 25-min reequilibration at 5%. The eluting peptides were interrogated with an Orbitrap Fusion mass spectrometer running a data-dependent LC-MS/MS method with the Top Speed decision selection. FTMS1 (Fourier Transform Mass Spectrometry) spectra were collected using the following parameters: scan range 350 to 1,800 *m/z*, resolving power 120k, automatic gain control (AGC) target 4E5, and maximum injection time of 50 ms. ITMS2 (Ion Trap Tandem Mass Spectrometry) spectra were collected using the following parameters: rapid scan rate, collision-induced dissociation (CID) NCE (normalized collision energy) 35, 1.6 *m/z* isolation window, AGC target 1E4, and maximum injection time of 50 ms. MS2 (Mass Spectrometry) precursors were selected for a 3-s cycle. Precursors with an assigned monoisotopic *m/z* and a charge state of 2 to 7 were interrogated. Precursors were filtered using a 60-s dynamic exclusion window.

### Phosphopeptide identification and protein analysis.

Raw mass spectrometric data were analyzed in the MaxQuant environment (version 1.6.0.16). The MS/MS spectra were matched against the Aspergillus fumigatus UniProt FASTA database. Enzyme specificity was set to trypsin, and the search included cysteine carbamidomethylation as a fixed modification and N-acetylation of protein, oxidation of methionine, and/or phosphorylation of Ser, Thr, and Tyr residues (STY) as variable modifications. Up to two missed cleavages were allowed for protease digestion, and peptides had to be fully tryptic.

The analyses of phosphopeptide, phosphorylation sites, and number of phosphoresidues were performed using filter tools in the software Microsoft Office Excel. Student’s *t* test (two-tailed, 95% [*P* < 0.05]) was performed for identification of differentially phosphorylated peptides between CR and T0 wild-type samples, CR mutant and CR WT samples, or T0 mutant and T0 wild-type samples. The total phosphopeptides identified were used for kinase site prediction by the software GPS 3.0, according to the default parameters, including medium threshold. For analysis of conservation between kinase family phosphosites, all sequences obtained from GPS software were selected for sequence logo building for the p38 MAPK family, using the software Weblogo (http://weblogo.berkeley.edu/logo.cgi), according to default parameters. All heatmaps were produced using Permutmatrix software (version 1.9.3.EN; http://www.atgc-montpellier.fr/permutmatrix/) according to default parameters, including Euclidean distances. Protein categorizations were performed by the FungiFun website (https://sbi.hki-jena.de/fungifun/) using A. fumigatus Af293 as species and identifiers (IDs) from each supplemental table (only “AFUA_” IDs were used).

### Vector construction for CRISPR-Cas9.

Vector construction was performed according to the methods described in reference 73. Primers and donor oligonucleotides (TAG Copenhagen, Copenhagen, Denmark) were designed using the A. fumigatus genomic sequence of each target (Afu6g12820_mpkB, Afu2g15010_srrB, and Afu6g04500_amk2; available on the Aspergillus Genome Database [AspGD], http://www.aspergillusgenome.org/) and are indicated in Table S8A and B in the supplemental material.

10.1128/mBio.02962-19.10 TABLE S8 (A) List of primers for “biobrick” amplification, fragment amplification, and Sanger sequencing. (B) List of donor oligonucleotides designed. The red nucleotides correspond to the codon to be edited, and the underlined nucleotides correspond to the point mutation. Download Table S8, DOCX file, 0.01 MB. Copyright © 2020 Mattos et al. 2020 Mattos et al. This content is distributed under the terms of the Creative Commons Attribution 4.0 International license.

The single guide RNAs (sgRNAs) were linked into the CRISPR-Cas9 vector pFC330 according to the tails of two primers. Previously, PCR fragments were amplified using the *Pfu* X7 polymerase with the primers from Table S8A and using pFC902 as a template. The “biobrick” fragments were further fused to the digested pFC330 by uracil-specific excision reagent (USER) fusion and USER cloning (74). Plasmids were purified with the GenElute plasmid miniprep kit (Sigma-Aldrich) and confirmed by restriction analysis. The selection of the transformants was performed using MM as selective medium, because the CRISPR-Cas9 vector (pFC330) contains *pyrG* as an auxotrophic marker.

### Protoplastization and genetic transformation.

Protoplastization of A. fumigatus was achieved using protocols previously described (73, 75). For transformation, 5 to 10 μl of the 100 μM stock donor oligonucleotide solution (Table S2) and 10 μg of the CRISPR-Cas9 vector were mixed with 50 μl protoplasts, 150 μl of PCT solution (50% [wt/vol] polyethylene glycol [PEG] 8000, 50 mM CaCl_2_, 20 mM Tris, 0.6 M KCl, pH 7.5) was added, and the sample was incubated at room temperature for 10 min. The mixture was then mixed with 250 μl ATB solution (1.2 M sorbitol, 50 mM CaCl_2_·2H_2_O, 20 mM Tris, 0.6 M KCl, pH 7.2). The protoplasts were plated on osmotic and selective TM medium and incubated at 37°C until transformants appeared on the transformation plates (approximately 1 week). Transformants were restreaked four times in MM, genomic DNA was extracted, and the PCR fragments for each gene were sent for DNA sequencing to confirm the presence of the respective mutation (the list of primers used for sequencing is in Table S8A).

### Protein-protein network analysis.

For network analysis, phosphoproteomic data were filtered for differences in phosphorylation level of >2-fold or <−2-fold and *P* value of >0.05. Next, data were filtered and analyzed through ad hoc R scripts (total number of differentially expressed phosphoproteins = 621) for the generation of Venn diagrams (VennDiagram package obtained from the following CRAN: https://cran.r-project.org/web/packages/VennDiagram/index.html) representing the intersections of the differentially phosphorylated proteins between the previously described studied strains. The list of differentially phosphorylated proteins and functional enrichment was obtained using the STRING server (https://string-db.org/), medium confidence threshold of 0.4 for interaction scores, using Aspergillus fumigatus as a model organism, and the generated functional protein association network presented a total of 336 nodes and 1,862 interactions as 26 phosphoproteins could not be found on the STRING server and the other 259 phosphoproteins did not present any association scores within the given set. The association network was then reprocessed through ad hoc R scripts for acquiring only “AFUA_” IDs for each of its nodes and was finally plotted/analyzed in Cytoscape software (https://cytoscape.org/). For network visualization, the fold change of phosphorylation levels was transformed to log_2_ and loaded in Cytoscape for color mapping as a heatmap scale (blue-white-red), and the node degree was mapped to node size. Node shapes were also mapped to protein functional domains representing three classes: kinases, phosphatases, and other protein domains.

### Sequence alignment and homology modeling procedures.

The search for *RCK1* homologues with solved structures deposited in the Protein Data Bank (PDB; http://www.rcsb.org) was performed using the web server BLASTp (Basic Local Alignment Search Tool, for proteins) algorithm (https://blast.ncbi.nlm.nih.gov/Blast.cgi?PAGE=Proteins). A protein with the PDB ID 3LIJ (crystal structure of full-length CpCDPK3 [cgd5_820] in complex with Ca^2+^ and AMP-PNP, at 1.9-Å resolution—a calcium/calmodulin-dependent serine/threonine kinase from Cryptosporidium parvum) was detected and here used as a template to build three homology models for *RCK1*, with respect to the wild-type residue (S481) and two mutations on residue 481 (S481A and S481D), using Modeller v. 9.2 (76). The pairwise alignments used as input for Modeller were previously performed using the web server Clustal Omega (v. 1.2.4; https://www.ebi.ac.uk/Tools/msa/clustalo/) and subsequently refined by structural inspection of the template, thus avoiding gaps (by deletion or insertion) inside secondary structure elements. Models were analyzed/validated regarding atomic contacts, chemical environment, and stereochemistry, using the web server SAVES v.5.0, at http://servicesn.mbi.ucla.edu/SAVES/.

### MD procedures.

Molecular dynamics (MD) calculations were carried out using the Biovia Discovery Studio software (77), founded on the CHARMm molecular mechanics force field engine. We used the standard dynamics cascade. Molecular dynamics was done with no restraints using as input the homology models built for *RCK1* (Afu2g03490), a calcium/calmodulin-dependent protein kinase from A. fumigatus, with respect to two mutations on residue S481 (S481A and S481D). At the first step, energy minimization was carried out using steepest descent with 100 maximum steps and a root mean square (RMS) gradient of 1.0. At the second step of the MD protocol, energy minimization was performed setting the conjugated gradient as algorithm, with 500 maximum steps and an RMS gradient of 0.1. For the heating step, we used a simulation time of 50 ps, with 2-ps time steps and initial and target temperatures of 50 and 300 K, respectively. The equilibration phase was carried out with 100 ps and target temperature of 300 K. On the production step, a simulation time of 1,000,000 ps (1,000 ns) was considered, saving results at 1,000-ps intervals (snapshots). We used an implicit solvent dielectric constant of 80 (simulation in water), with a nonbond higher cutoff distance of 12.0 and nonbond lower cutoff distance of 10.0, with a leapfrog Verlet dynamics integrator.

### Phenotypic characterization of A. fumigatus null mutant strains.

A. fumigatus conidia (10^5^) of wild-type (CEA17) and null mutant strains (Table 2) were inoculated on solid minimal medium (MM; 1% glucose, 6 g/liter NaNO_3_, 0.52 g/liter KCl, 1.52 g/liter KH_2_PO_4_, 0.52 g/liter MgSO4·7H_2_O, 1 ml/liter of trace elements, pH 6.5, 2% agar) with different concentration of CR, CFW, sorbitol, and caspofungin and grown for 5 days at 37°C. The radial growth of each strain in the presence of the molecules was normalized by the growth of each strain in MM without any molecule. All plates were grown in triplicate, and the average ± SD was plotted. A Student *t* test using Prism GraphPad (version 6) was applied to confirm the statistical significance difference between treatment and control (*P* value < 0.05).

10.1128/mBio.02962-19.2 FIG S2 Heatmap of the phosphorylation profile of the 16 phosphoproteins (log_2_ fold change) shared between all strains under CR exposure. It is clear that, although ubiquitous in the CR stress response, they are differentially phosphorylated according to the strain genetic background. Download FIG S2, PDF file, 0.03 MB. Copyright © 2020 Mattos et al. 2020 Mattos et al. This content is distributed under the terms of the Creative Commons Attribution 4.0 International license.
