# Supplementary material for: The Aspergillus fumigatus Phosphoproteome Reveals Roles of High-Osmolarity Glycerol Mitogen-Activated Protein Kinases in Promoting Cell Wall Damage and Caspofungin Tolerance
Source: mBio. 2020 Feb 4;11(1):e02962-19. doi: 10.1128/mBio.02962-19 (PMC7002344; doi:10.1128/mBio.02962-19)
Supplement: FIG S1 [file mBio.02962-19-sf001.pdf]

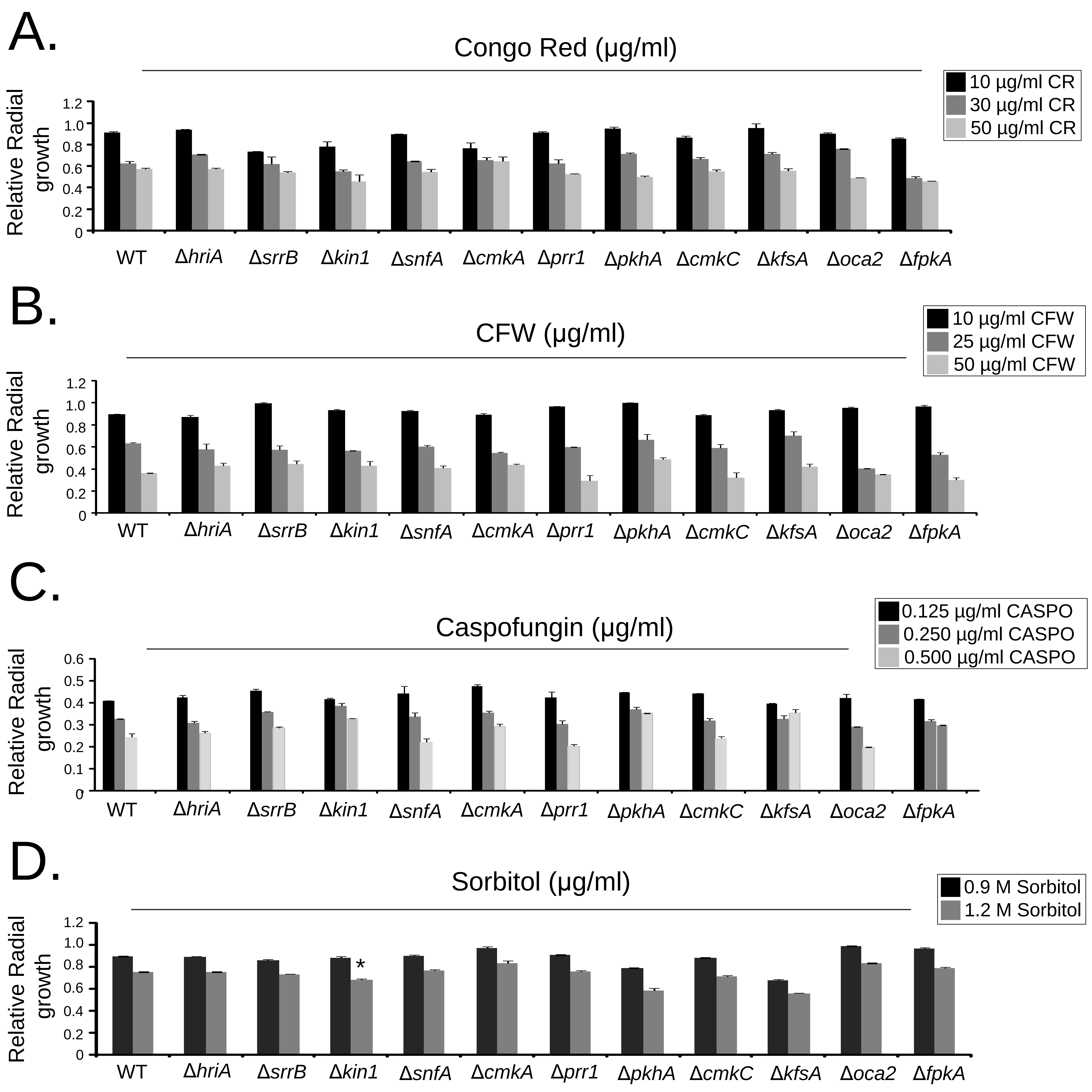
A.
Congo Red (μg/ml)
10 μg/ml CR
30 μg/ml CR
50 μg/ml CR
Relative Radial growth
1.2
1.0
0.8
0.6
0.4
0.2
0
WT
ΔhriA
ΔsrrB
Δkin1
ΔsnfA
ΔcmkA
Δprr1
ΔpkhA
ΔcmkC
ΔkfsA
Δoca2
ΔfpkA
B.
CFW (μg/ml)
10 μg/ml CFW
25 μg/ml CFW
50 μg/ml CFW
Relative Radial growth
1.2
1.0
0.8
0.6
0.4
0.2
0
WT
ΔhriA
ΔsrrB
Δkin1
ΔsnfA
ΔcmkA
Δprr1
ΔpkhA
ΔcmkC
ΔkfsA
Δoca2
ΔfpkA
C.
Caspofungin (μg/ml)
0.125 μg/ml CASPO
0.250 μg/ml CASPO
0.500 μg/ml CASPO
Relative Radial growth
0.6
0.5
0.4
0.3
0.2
0.1
0
WT
ΔhriA
ΔsrrB
Δkin1
ΔsnfA
ΔcmkA
Δprr1
ΔpkhA
ΔcmkC
ΔkfsA
Δoca2
ΔfpkA
D.
Sorbitol (μg/ml)
0.9 M Sorbitol
1.2 M Sorbitol
Relative Radial growth
1.2
1.0
0.8
0.6
0.4
0.2
0
*
WT
ΔhriA
ΔsrrB
Δkin1
ΔsnfA
ΔcmkA
Δprr1
ΔpkhA
ΔcmkC
ΔkfsA
Δoca2
ΔfpkA

Supplementary Figure S1. Null kinase mutants grown on different stress conditions. *A. fumigatus* Conidia (10^5) were inoculated on solid minimal medium (MM) with different concentrations of Congo Red, Calcofluor white (CFW), Sorbitol and caspofungin and grown for 5 day at 37°C.
