# Supplementary material for: The Aspergillus fumigatus Phosphoproteome Reveals Roles of High-Osmolarity Glycerol Mitogen-Activated Protein Kinases in Promoting Cell Wall Damage and Caspofungin Tolerance
Source: mBio. 2020 Feb 4;11(1):e02962-19. doi: 10.1128/mBio.02962-19 (PMC7002344; doi:10.1128/mBio.02962-19)
Supplement: TABLE S8 [file mBio.02962-19-st008.docx]

**Supplementary Table S8A. List of primers for “biobrick” amplification and for fragment amplification and Sanger sequencing.**

| **Sequence name** | **Sequence 5' → 3'** |
| --- | --- |
| Afu6g12820_mpkB_Fw | ACGTTGCCACUCGAGTTTTAGAGCTAGAAATAGCAAG |
| Afu6g12820_mpkB_Rv | AGTGGCAACGUATTCCGTGCATCATCCGTGAATCGAAC |
|  |  |
| Afu2g15010_srrB_Fw | ACGATCGGATCUCAAGAGTTTTAGAGCTAGAAATAGCAAG |
| Afu2g15010_srrB_Rv | AGATCCGATCGUCTGTGCATCATCCGTGAATCGAAC |
|  |  |
| Afu6g04500_amk2_Fw | AACCAACGCAAUATCGTTTTAGAGCTAGAAATAGCAAG |
| Afu6g04500_amk2_Rv | ATTGCGTTGGTUGACGGTGCATCATCCGTGAATCGAAC |
|  |  |
| Afu3g11080_bckA_Fw | ATGAAGATUCGGACGAGTTTTAGAGCTAGAAATAGCAAG |
| Afu3g11080_bckA_Rv | AATCTTCAUCGGATGCATCATCCGTGAATCGAAC |
|  |  |
| CSN438 (Nødvig *et al.*, 2018) | GGGTTTAAU GATCACATAGATGCTCGGTTGACA |
| CSN790 (Nødvig *et al.*, 2018) | GGTCTTAAU ACCCTGAGAAGATAGATGTGAATGTG |
|  |  |
| Afu6g12820_503_Fw | TGGACAGGAGTTGATGGA |
| Afu6g12820_503_Rv | TCTTCCATGGTTGGTGTT |
|  |  |
| Afu6g04500_503_Fw | TTATCTACTTTGCGCTTG |
| Afu6g04500_503_Rv | GAGAAACTGGTACTTGCA |
|  |  |
| Afu2g15010_512_Fw | TCTTGTGCAATTCAGTAC |
| Afu2g15010_512_Rv | CCATCCTGGAAACCGGAT |
|  |  |
| Afu3g11080_515_Fw | CTTTCGTTTCTTTCCTTC |
| Afu3g11080_515_Rv | GTGAAGCGGCCATGAGGA |

**Supplementary Table S8B. List of donor oligonucleotides designed.** The red nucleotides correspond to the codon to be edited, and the underlined nucleotide correspond to the point mutantion.

| **Sequence name** | **Sequence 5' 🡪 3'** |
| --- | --- |
| Afu6g12820_**mpkB**_184Phe | GTCGGCTGCATCGACTGATGACAACTCAGGTTTCATGACGGAA**TTC**GTTGCCACTCGATGGTATCGTGCGCCGGAGATCATGCTGACAT |
|  |  |
| Afu6g12820_**mpkB**_184Asp | GTCGGCTGCATCGACTGATGACAACTCAGGTTTCATGACGGAA**GAC**GTTGCCACTCGATGGTATCGTGCGCCGGAGATCATGCTGACAT |
|  |  |
| Afu2g15010_**srrB**_1466Ala1469Ala | CCATCGACAGGGCAGTATCCCAACGACCCGAGCGAGATCTCAG**GCG**ATCGGA**GCT**CAAGATGGAGAATTTTCGTCCTCTCTCGCAAAGGAATCGTATG |
|  |  |
| Afu2g15010_**srrB**_1466Asp1469Asp | CCATCGACAGGGCAGTATCCCAACGACCCGAGCGAGATCTCAG**GAT**ATCGGA**GAT**CAAGATGGAGAATTTTCGTCCTCTCTCGCAAAGGAATCGTATG |
|  |  |
| Afu6g04500_**amk2**_36Ala | TTTATCGGCTCCCGCAGGCGATAAGAAGGTGAATCGCCGGCCGGCAACCAACGCAATATCCGGGGCAGCAAAGGCAACTGCGGCCGACC |
|  |  |
| Afu6g04500_**amk2**_36Asp | TTTATCGGCTCCCGCAGGCGATAAGAAGGTGAATCGCCGGCCGGACACCAACGCAATATCCGGGGCAGCAAAGGCAACTGCGGCCGACC |
|  |  |
| Afu3g11080_**bckA**_1039Ala1043Ala | GACTGAAGTCTCCTTCCAACGCTCGCCCAGGCCACAGGATGAT**GCC**GATGAAGAT**GCC**GACGATGGTCTATTTGCGATACCGCTGTCAAATAATAAAGCAT |
|  |  |
| Afu3g11080_**bckA**_1039Ala1043Ala | GACTGAAGTCTCCTTCCAACGCTCGCCCAGGCCACAGGATGAT**GAT**GATGAAGAT**GAT**GACGATGGTCTATTTGCGATACCGCTGTCAAATAATAAAGCAT |
