# Supplementary material for: The Aspergillus fumigatus Phosphoproteome Reveals Roles of High-Osmolarity Glycerol Mitogen-Activated Protein Kinases in Promoting Cell Wall Damage and Caspofungin Tolerance
Source: mBio. 2020 Feb 4;11(1):e02962-19. doi: 10.1128/mBio.02962-19 (PMC7002344; doi:10.1128/mBio.02962-19)
Supplement: FIG S2 [file mBio.02962-19-sf002.pdf]

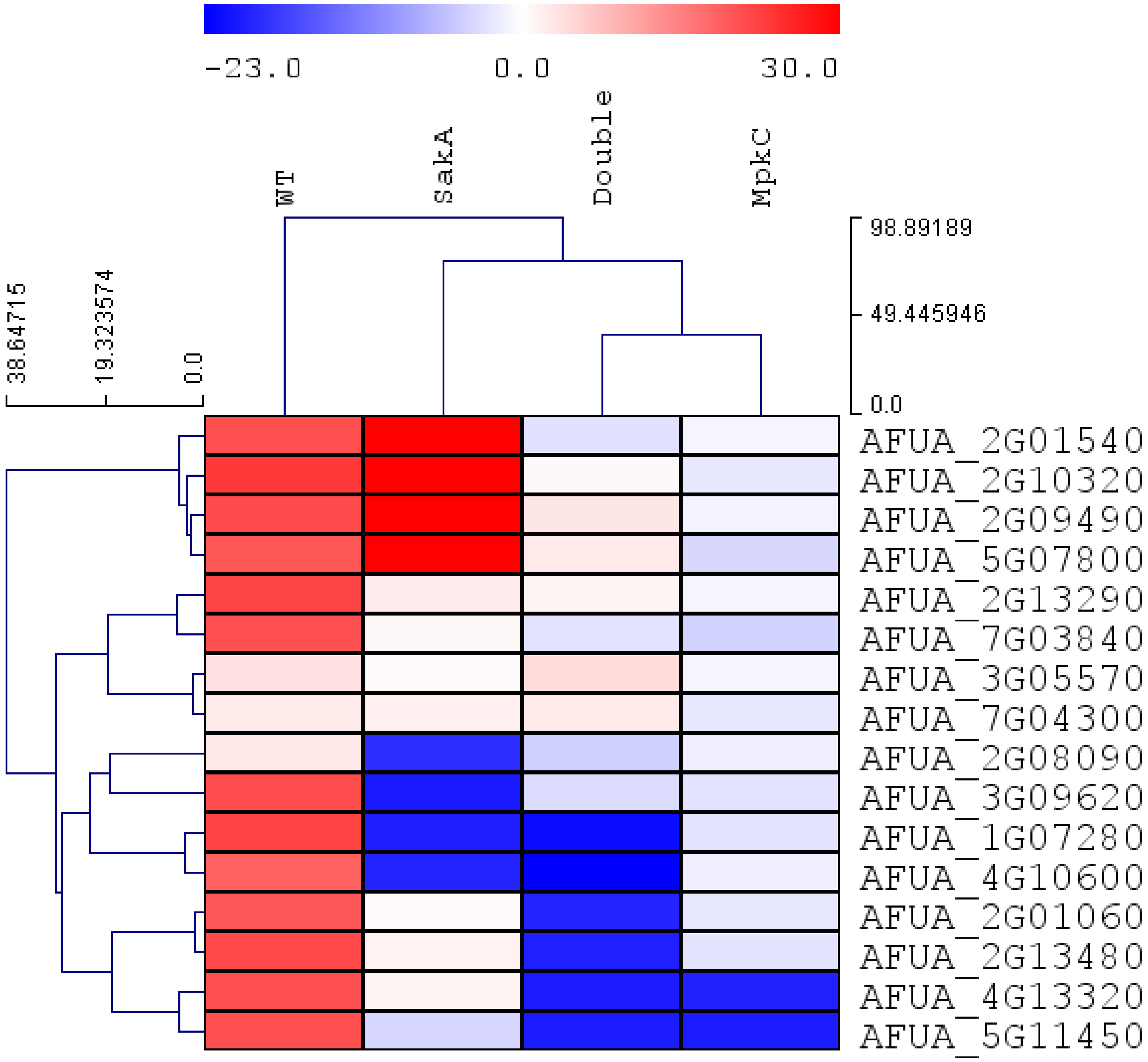

Supplementary Figure S2 - Heatmap of the phosphorylation profile of the 16 phosphoproteins (log2 fold-change) shared between all strains under CR exposure. It is clear that, although ubiquitous in the CR stress response, they are differentially phosphorylated according to the strain genetic background.
